# Combating Bacterial Resistance by Polymers and Antibiotic Composites

**DOI:** 10.3390/polym16233247

**Published:** 2024-11-22

**Authors:** Iulia Olaru, Alina Stefanache, Cristian Gutu, Ionut Iulian Lungu, Cozmin Mihai, Carmen Grierosu, Gabriela Calin, Constantin Marcu, Tudor Ciuhodaru

**Affiliations:** 1 Faculty of Medicine and Pharmacy, University “Dunarea de Jos”, 47 Domneasca Str., 800008 Galati, Romania; 2 Faculty of Pharmacy, “Grigore T. Popa” University of Medicine and Pharmacy, 700115 Iasi, Romania; 3 Faculty of Dental Medicine, “Apollonia” University of Iasi, 11 Pacurari Street, 700115 Iasi, Romania

**Keywords:** antibiotic resistance, polymer, biopolymers, phosphonium salt, nanoparticles

## Abstract

(1) Background: Since the discovery of antibiotics in the first half of the 20th century, humans have abused this privilege, giving rise to antibiotic-resistant pathogens. Recent research has brought to light the use of antimicrobial peptides in polymers, hydrogels, and nanoparticles (NPs) as a newer and safer alternative to traditional antibiotics. (2) Methods: This review article is a synthesis of the scientific works published in the last 15 years, focusing on the synthesis of polymers with proven antimicrobial properties. (3) Results: After a critical review of the literature was made, information and data about the synthesis and antimicrobial activity of antibacterial polymers and NPs functionalized with antibiotics were extracted. Fluorinated surfactants such as the Quaterfluo^®^ series presented significant antimicrobial effects and could be modulated to contain thioesters to boost this characteristic. Biopolymers like chitosan and starch were also doped with iodine and used as iodophors to deliver iodine atoms directly to pathogens, as well as being antimicrobial on their own. Quaternary phosphonium salts are known for their increased antimicrobial activity compared to ammonium-containing polymers and are more thermally stable. (4) Conclusions: In summary, polymers and polymeric NPs seem like future alternatives to traditional antibiotics. Future research is needed to determine functional doses for clinical use and their toxicity.

## 1. Introduction

The turn of the last century has seen the emergence of antibiotics to treat infections that until then could be deadly, reducing those disease-causing pathogens to mere inconveniences. Even if today we regard a bacterial infection as a mundane thing requiring a day off and a prescription from the doctor, people in the early 1900s were not so lucky; for them, a simple cut while performing everyday tasks could lead to severe infections [1]. In those cases, most of the time, sulfonamides would be given to the patient as a way of inhibiting the growth of bacteria. Even though they are an important class of medication and are still used today to treat bacterial infections [2], obesity [3], and thyroid problems [4], back then sulfonamides would often prove to be ineffective. In the unfortunate scenario when the patient would not respond to the sulfonamide treatment, amputation would be the next step; in the worst case, the individual would succumb to a painful and slow death.

Alexander Fleming is credited with the discovery and extraction of penicillin, which he came across in his contaminated bacterial colonies after returning from a trip. Upon noticing that a mold from the *Penicillium* genus has inhibited the growth of bacteria on the agar substrate, he deduced that the fungi produced a chemical that stumped the growth of Gram-positive bacteria and staphylococci. However, it would take more than a decade for a team at Oxford University to isolate and treat mice infected with a viral species of *Streptococcus* [5]. In the 1970s, a group of researchers continued studies on the mechanism of action of penicillin, discovering a chemical and structural similarity between penicillin and the D-Ala-D-Ala ending of the peptidoglycan (PGN) of Gram-positive and Gram-negative pathogens’ cell walls [6]. The PNG or murein is a heteropolymer that forms a macromolecule that protects the cytoplasm of pathogens, conferring rigidness and stability to the cell wall [7].

The biosynthesis of PGN, like in many other Gram-negative bacteria, is divided into two distinct steps. The synthesis starts inside the cytoplasm, where it is catalyzed by murA. UDP-N-acetylmuramate is produced from the enol-pyruvate moiety that is reduced to D-lactate; this reaction is further catalyzed by murB. UDP-N-acetylmuramate further enters a series of pentapeptide side chain additions. The second step is key to the formation of the cell wall, transporting the precursor across the cell membrane, fortifying it, and adding it to the other present polymers. Further tests have deemed that the administration of antibiotics has limited to no effect on mur enzymes [8]. However, the UDP-N-acetylglucosamine-3-enolpyruvyltransferase or murA enzyme is targeted by a phosphonic broad-spectrum antibiotic, fosfomycin, which impedes the normal function of the enzyme, causing an unregulated synthesis of the cell wall, resulting in elongated cells [9].

The discovery of penicillin gave rise to the antibiotic class of β-lactams, which over the years expanded into other subgroups and derivatives such as monobactams, cephalosporins, and carbapenems which aimed to address a wider range of bacterial species and infections. β-lactams constitute over 65% of the prescribed antibiotics in the US, with cephalosporins representing the majority of them [10]. Ever since antibiotics were officially introduced in the 1940s, their use and misuse gave rise to what some experts call a crisis of the 21st century, that being antibiotic resistance [11].

## 2. Mechanism of Antibiotic Resistance

**Figure 1:**
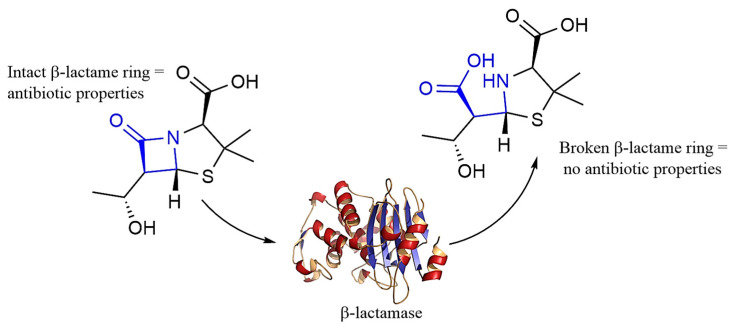
β-lactamase plays a crucial role in the resistance of pathogens; it acts by breaking open the β-lactam ring (blue), inactivating the antibiotic.

Antibiotic resistance and antibiotics are not a recent discovery, they predate humans and the controlled medication of bacterial infections. Class A β-lactamases are ancient antibiotic-resistant enzymes that can cleave the β-lactam ring (Figure 1), rendering the antibiotic useless. These enzymes developed over billions of years, being later transferred to Gram-positive pathogens [12,13].

Resistance to antibiotics has led to the emergence of superbugs and pathogens resistant to one or more antibiotics no matter the concentration. As it is with every living being, bacteria can evolve and during this development, its genes can mutate, acquiring resistance; it does not help that the misuse of antibiotics is monumental in this development and can deem otherwise broad-spectrum, functional antibiotics useless [14]. Research has also found that the abuse of antimicrobials in animal farms can give rise to superbugs or DNA fragments that can encode resistance which can be transmitted from an animal host to a human one [15].

In this fight against humans and bacteria, antibiotics are deemed useless by the rapid development of these organisms, this is why we must find other alternatives to which bacteria cannot form an immunity [16]. Polymers and polymeric NPs seem to be the best candidates, thanks to their ability to mimic natural antimicrobial peptides (AMPs) and carry antibiotics, bypassing the pathogens’ innate immunity, making them one of the best alternatives [17,18].

The misuse of antibiotics and antibiotic resistance of pathogens are considered threats to ecological systems and public health, turning bacteria that would have otherwise been treated with a common antibiotic into shielded organisms [19].

Multidisciplinary research in medicine, pharmacy, and physics is necessary for the development of newer and better antimicrobial agents that can get past the defenses of Gram-positive and Gram-negative bacteria by disrupting biofilm formation and by denaturing resistance enzymes such as β-lactamases [20]. Bacteria possess a plethora of defense mechanisms such as efflux pumps; these modified structures evolved along the ejection of antimicrobials outside the pathogen. Efflux pumps are the major reason Gram-negative pathogens resist antibiotics that would otherwise be lethal to Gram-positive ones [21]. It was found that antibiotics, which are positively charged, bind to the negatively charged polymers found in the matrix of biofilm, stopping the absorption of antibiotics like aminoglycosides [22].

Further research brought to light the fact that diverse and complex biofilms formed from different microbial species are more resistant to antibiotics than the ones formed by a single strain. At the same time, cells that lay dormant in the biofilm’s layers are often immune to antibiotics such as aminoglycosides, β-lactams, and fluoroquinolones [23,24].

## 3. Progress of Bacterial Resistance Polymers and Polymeric Composites

Polymers are complex macromolecules that can be modulated to any need; in combating antibiotic resistance and pathogens, medical devices such as catheters and stents can be coated with these polymers to prevent biofilm formation and the adherence of bacterial colonies. Polymers can also be used to mimic antibacterial AMPs or can be integrated into hydrogels and NPs [25].

### 3.1. Bacterial Resistance Polymers

In recent years, polymer antibiotic hydrogels have been developed for clinical use in treating bacterial infections. Hydrogels are 3D nanostructures formed from a complex matrix of intertwined polymer chains that are bound to each other either physically or thought chemical means. They are lightweight, porous materials that have found many uses in the field of biomedicine, drug delivery, NP synthesis, and so much more [26,27].

Antimicrobial peptides (APs) are a group of peptides of the innate immune system and are constituted of up to fifty amino acids. These peptides are amphiphilic with a positive charge thanks to arginine and lysine residues, which allow them to deposit themselves on the bacterial membrane and form pores, killing the pathogen [28]. APs can be used and modulated into antimicrobial polymers that can get over the resistance to traditional antibiotics. These peptides are broad-spectrum and offer defense against both Gram-positive and Gram-negative bacteria, viruses, and fungi. At this moment, there have been discovered and documented four groups of peptides: β-sheet peptides (e.g., human α- and β-defensins [29]), loop peptides, extended structures that are rich in tryptophan or serine, and α-helical peptides [30].

#### 3.1.1. Halogen-Containing Polymers

At this moment, the literature is filled with a variety of polymers used to combat antibiotic resistance; for example, halogen (fluorine, bromine, chlorine, and iodine)-containing polymers follow the general structure of antibiotics like chloramphenicol (containing chlorine) or fluoroquinolones (containing fluorine). Recent studies have shown that halogenated polymers exhibit enhanced antibacterial activity by disrupting bacterial membranes, preventing biofilm formation, and inhibiting bacterial growth, even in resistant strains such as MRSA and P. aeruginosa strains resistant to multiple antibiotics. These polymers have been shown to penetrate bacterial cell walls and interact with essential bacterial proteins, leading to reduced resistance mechanisms over time [31,32].

**Figure 2:**
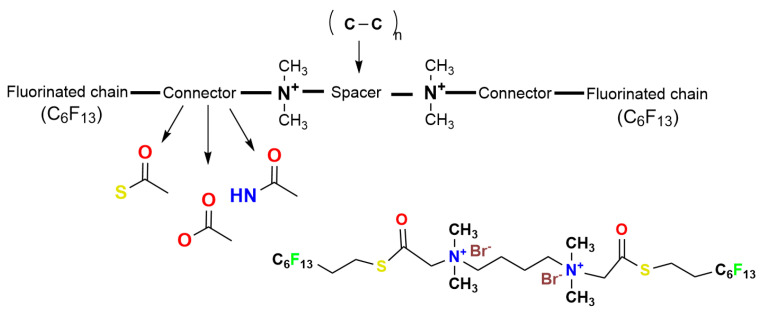
General Quaterfluo^®^ surfactants structure and Quaterfluo^®^ C3.

These halogen polymers are popular because of their hydrophobic properties, making them ideal against resistant pathogens such as *S. aureus* and *P. aeruginosa*. Quaterfluo^®^ is a surfactant synthesized from the bonding of fluoroalkylated radicals to an ammonium salt structure. The general structure of these polymers (Figure 2) revolves around alkylated quaternary ammonium salts which are bound together by so-called “connectors”, such as ester, amide, or thioester groups. The connecters bind the ammonium salts to a chain of fluorinated carbons [33].

**Figure 3:**
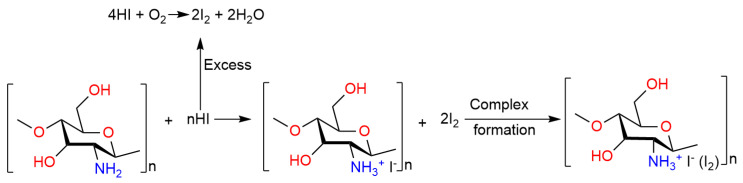
The synthesis of an iodine-doped chitosan complex.

Prior research on the generation of Quaterfluo^®^ Tx has revealed that T4 was the most efficient at combating Gram-negative bacteria. The T4 compound synthesized by researchers follows the general structure presented in Figure 3. with a six-carbon bridge binding the two ammonium cations. The same group of researchers has determined that a longer spacer increased effectiveness against strains such as *S. aureus* and *C. albicans*. Quaterfluo^®^ T4 has a unique design by the employment of thioester groups in the structure of the surfactant [31]. Thioesters are known for their antibacterial properties and they have proven themselves as inhibitors of metallo-β-lactamase L1, combating antibiotic resistance [34,35].

Biopolymers have become more popular in recent years thanks to the spotlight that has been put on green synthesis, and their biodegradable, non-toxic, and biocompatible properties. Chitosan is one of these biopolymers; it is a synthetic molecule derived from the chitin found in the exoskeleton of crustaceans [36]. Chitosan has been proven to be effective in combating bacteria by causing the depolarization of the bacterial membrane through the displacement of divalent metal ions. Chitosan is successful against Gram-negative pathogens because it interacts with lipopolysaccharides; studies on high-molecular-weight chitosan have proven its ability to engulf pathogens, slowly destroying and rupturing the membrane of bacteria [37,38].

Table 1 describes the main mechanisms of action in which chitosan molecules act against bacteria, especially Gram-negative strains. Because of its complex structure, chitosan is often used in the field of nanotechnology, especially in NPs with different functions such as antibacterial ones or in drug delivery systems. Chitosan is also used in tissue engineering in the form of 3D scaffolds thanks to their porosity which offers structure for new cell growth, replacing diseased or dead tissue [39].

Thanks to its base antibacterial properties, chitosan is an ideal building block in the synthesis of new antibiotic substances. Halogen-containing complexes need a backbone structure to be delivered and released from for medication stability; starch and chitosan already present antibacterial properties and are ideal for this.

Edis et al., 2019 reported that the long-term stability of the biocidal agent is important for the steady release of I_2_ [49]. On the same theme of iodine–chitosan derivatives, a group of researchers managed to synthesize a hydroxypropyl chitosan biguanide hydrochloride polyvinylpyrrolidone iodine membrane (HPCGH-PVP-I_2_-M); this polymer released iodine at a constant speed that increased with the rise in temperature [50].

HPCGH-PVP-I_2_-M was found through the inhibition zone diameter test to be a more effective antibiotic than its prior reagents, with 28 ± 1 mm for *E. coli* and 30 ± 1 mm for *S. aureus*. The same group of researchers tested the release of iodine from the membrane over a period of 200 min at different temperatures and they showed that an increase in temperature leads to the excitement of iodine and release of it from the membrane into the buffer solution [51].

The results of the iodine release test are shown in Table 2.

The study of chitosan being used as an iodophor is still limited but seems to catch more interest from researchers as antibiotic resistance soars on and traditional antibiotics are slowly replaced by greener and much more effective alternatives. Iodine–chitosan compounds are mainly present as membranes and films and their synthesis can be as simple or as complicated as they need to be for the final product to have high specificity for different bacterial proteins and enzymes. For the synthesis of these substances, the chitosan is either treated with HI, elemental iodine, or a mixture of iodine and potassium iodide; all these methods yield chitosan polymers doped with iodine and iodide ions that bind to the nitrogen atoms [52]. Figure 3. depicts the synthesis process where chitosan is used as an iodophor, and the final product is an iodine–polysaccharide complex, where the initial amine group has been turned into an ammonium quaternary salt which has proven antibacterial properties. Together with iodine, this complex plays a pivotal role in antibiotic and polymer research, having been modulated to be an effective antibacterial weapon [53,54,55].

Electrochemistry plays a crucial role in the synthesis of chlorinated chitosan films for wound protection and healing. Qu et al., 2018 reported the electro-fabrication of a chloramine-based membrane directed towards wound healing. The making of this film was simple: Firstly, the researchers started by dissolving chitosan in a HCl solution, reaching a desired pH of around 5 to 6. After this, a sheet electrode made from platinum was immersed in the solution and a cathodic current was applied to it; this led to the deposition of chitosan out of the solution onto the platinum plate.

After this step, the sheet containing the chitosan film was washed with water and placed in a phosphate-buffered solution with a pH of 7 and 0.5 NaCl for the chloride anions. The chlorination of the chitosan film was induced by an anodic voltage of 3V. After the electrochemical process, the simple chitosan and chitosan–chlorine films were analyzed using different techniques and equipment. Scanning electron microscopy (SEM) revealed that the two films were morphologically different with the chitosan film having a smooth surface and being perfectly transparent, while the chitosan–chlorine film presented a rough and porous surface with bumps and was cloudy.

Furthermore, in vivo studies conducted by the researchers revealed that the chlorinated film was effective at healing a wound model by approximately 30% compared to the standard, and that the film inhibited the growth of methicillin-resistant *Staphylococcus aureus* (MRSA) [56].

#### 3.1.2. Phosphorus-Containing Polymers

Phosphonium-containing polymers are macromolecules that contain one or more phosphorus atoms in their structure; they are typically similar in structure to quaternary ammonium salts but have been proven to be much more effective than them at damaging the bacterial cell wall and disrupting the formation of biofilm [28,57].

Quaternary phosphorus salts (QPSs) have been widely used and researched since their discovery; these polymers are polycationic and can be alkylated or the QPS can be polymerized with natural macromolecules. Because of their cation structure, it is theorized that both ammonium and phosphorus quaternary salts act on the cell wall, penetrating it and getting into the matrix of the bacterial membrane where they act as surfactants, breaking the phospholipid layer, and leading to the leakage of the bacteria’s cytoplasm [58].

**Figure 4:**
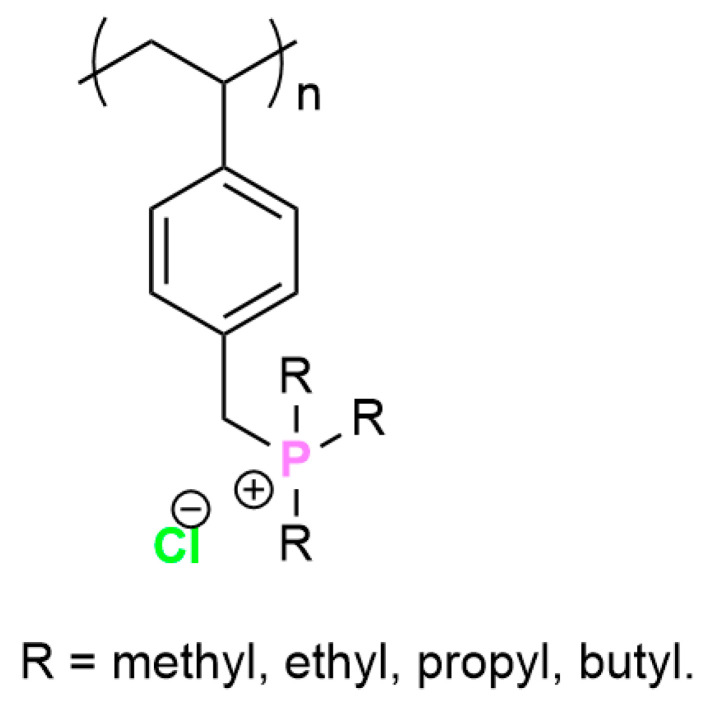
Phosphonium polystyrene polymer with different alkyl substituents.

QPS are less toxic than their ammonium counterpart, even though it is a mystery that there are so few publications reporting the synthesis and testing of QPS. These phosphonium salts are also more stable at different temperature shifts and do not degrade easily. This is confirmed by Hemp et al., 2013 [59] who synthesized ammonium and phosphonium polymerized ionic liquids with different alkyl substituents, after which they tested their respective thermal stability and ion conductivity. The structure of the phosphonium polymer is described in Figure 4.

The ammonium polymers containing chloride anions could not be tested because of their poor thermal stability and degradation below the glass transition temperature (T_g_). The researchers found that the phosphonium polymers had better thermal stability than their ammonium counterparts; they also found that increasing the length of the alkyl radicals decreased the heat stability of the polymers (Table 3) [59].

The increased biocidal ability of phosphonium salts and their respective polymers is thanks to the difference in the positively charged phosphonium ion compared to the ammonium one. The phosphorus cation is bigger and has a greater charge density compared to the nitrogen one; this character impacts the way biomolecules and membranes react to them [60,61,62,63].

The synthesis of organic QPS-containing polymers follows a straightforward method used widely by researchers. Most of the time, these polymers are synthesized from acrylate and methacrylate monomers. Monomers such as 4-vinylbenzyl(trimethyl phosphonium) chloride or 4-vinyl benzoic acid can be homo- and copolymerized by reversible addition–fragmentation chain transfer (RAFT). In this method, a transfer agent that is soluble in water such as trithiocarbonate (2-(2-carboxyethylsulfanylthiocarbonylsulfanyl) propionic acid) is used [64,65,66].

A handful of studies reported that alkyl (methyl, ethyl, propyl, and butyl) substituents in QPS polymers presented a lesser antimicrobial activity and did not cause significant hemolysis of red blood cells, while the aromatic ones were more toxic but presented higher antimicrobial activity against Gram-negative pathogens [57,67,68]. Phosphonium peptides synthesized from lab-designed amino acids derived from cysteine showed a decreased antimicrobial activity compared to AMP HHC10, but were less toxic and had the ability to cross cell membranes [69,70].

**Figure 5:**
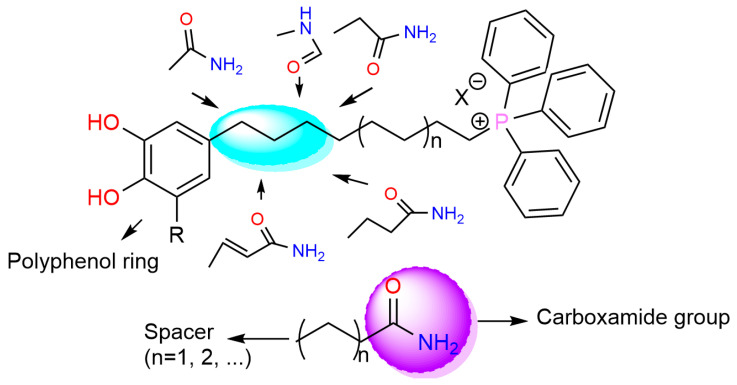
The architecture of triphenyl phosphonium salts combined with plant polyphenols.

A study by Chavarria et al., 2023 focused on the synthesis of triphenyl phosphonium salts conjugated with phytochemicals and their use against Gram-positive bacteria (MRSA); Gram-negative ones—*E. coli*, *K. pneumoniae*, *P. aeruginosa*, and *A. baumannii;* and fungi—*C. albicans* and *C. neoformans* var. *grubii*. The synthesized compounds followed a general structure, depicted in Figure 5.

The researchers found that derivatives with a ten-carbon chain linking the carboxamide group and the phosphonium salt had an increased antibacterial action compared to other alkyl radicals. The particular action towards *S. aureus* was maintained when methylene and ethylene radicals were used; besides this, they also made the compounds safer by being less toxic.

Hemolytic and cytotoxic effects were observed after the introduction of a vinyl spacer; this made the antibiotic substance more lipophilic and selective towards fungi but also more toxic. Even though the polyphenols used did not impact the antimicrobial activity significantly, in one exception, *C. neoformans* var. *grubii* was much more susceptible to pyrogallol derivatives than the catechol ones. It is believed that these synthesized compounds act by disrupting the potential of the bacterial membrane, causing deformation and in the end rupturing it, leading to leakage of the bacteria’s contents [67].

Nemeş et al., 2022 worked on the synthesis of cellulose and chitosan polymers functionalized with dodecyl-triphenyl phosphonium bromide. They found though microbiological tests that both the chitosan and cellulose functionalized polymer had a 100% inhibition against both Gram-positive (*S. aureus*) and Gram-negative (*E. coli*) bacteria. Regardless of the ratio used in the case of *P. aeruginosa*, limited antimicrobial activity was registered at a maximum of 46.6%. The increase in the functionalization rate was better in the case of the chitosan functionalized polymer increasing from 31% to 46.6% while in the case of cellulose, an increase of 7.8% was observed [71].

Despite the hydrophobic character of dodecyl-triphenyl phosphonium bromide, it was unable to completely penetrate the cell wall of *P. aeruginosa*, impeding its biocidal properties [72,73,74]. Dodecyl-triphenyl phosphonium bromide may also be attracted by lipids on the surface of the bacterial cell membrane through strong electrostatic forces, leading to cell death [75].

Triphenyl phosphonium salts have also been used against nosocomial infections, being tested for their use in clinical settings. QPS proved effective against one of the major staphylococci species in hospital environments, MRSA, which is the main pathogen associated with central line bacteremia. In the United States of America, MRSA is the most common cause of endocarditis [76]. Infective endocarditis is often related to the formation of biofilm and is one of the most common bacterial heart infections, leading to valvular vegetations and inflammations [77]. The biofilm structures are often formed by *S. aureus* and *epidermidis* which impede the penetration of antibiotics like vancomycin and cefotaxime; *Candida albicans* mixed biofilms also hinder the penetration of antifungals like fluconazole. Mupirocin encapsulated in NPs has been proved to be very effective in animal models, treating over 50% of the infected individuals compared to 0% in the individuals treated with free mupirocin [78]. Another study focused on bacterial colonization-prone pyrolytic carbon used in artificial heart valves; the researchers modified the surface of this polymer with 30 nm silver NPs and found that the functionalized material was not only non-toxic but also proved effective against MRSA [79].

A recent study looked at the activity and synergism between ammonium and phosphonium quaternary salt-containing polymers and found that poly(tributyl(4-vinylbenzyl)ammonium chloride and poly(tributyl(4-vinylbenzyl)phosphonium chloride (1:1) exhibited increased antimicrobial properties compared to individual ammonium and phosphonium polymers. It is believed that the bacterial membrane components are more soluble in the mixture of the two polymers, leading to better results [58].

### 3.2. New Antibiotic/Polymer Composite

#### 3.2.1. Halogen-Containing Antibiotic/Polymer Composite

NP and polymer architectonics offer researchers and the pharmaceutical industry almost unlimited opportunities for creating novel molecules with new and improved effects [80].

As the first part focused on the halogenation of chitosan chains in order to increase the antibacterial properties of both reagents, this part looks at the encapsulation of the existing halogen-containing antibiotics as a new way of delivering them to the targeted region.

Fluoroquinolones, glycopeptides, and lincosamides are halogen-containing antibiotics that have been used in recent research on antibiotic delivery systems. Chloramphenicol has also been used in NPs, but due to its haemotoxicity and its causing aplastic anemia, its use is limited to eye infections such as conjunctivitis and ear infections [81].

Hadiya et al., 2018 managed to synthesize levofloxacin-loaded chitosan NPs via ionic gelation and tested them on different *E. coli* mutated generations. Most resistance to fluoroquinolones appears due to mutations in the regulators of the efflux pump. The researchers discovered that the synthesized levofloxacin-loaded NPs were stable even after being frozen without the need for any cryoprotectant; they found that the solution containing the NPs could be freeze-dried and stored, making them ideal for clinical use. The NPs have also been proven to be more effective than traditional levofloxacin being more biocompatible [82].

López-López et al., 2019 employed a similar encapsulation method as the one above by using ionic gelation to encapsulate the antibiotic in NPs. However, they also tried a modified method: polyelectrolyte complexation with ionic gelation and added carrageenan (red seaweed polysaccharide) together with the chitosan. This slight change increased the ability of the base chitosan NPs, making them more effective at encapsulating levofloxacin, which they later release [83].

Another group of researchers made chitosan NPs based on sulfobutyl-ether-*β*-cyclodextrin (SBE-*β*-CD) for the delivery of levofloxacin to treat bacterial ocular infections. Further spectroscopy tests such as UV-Vis and 2D NMR revealed that SBE-*β*-CD is ideal because it forms a 1:1 complex with levofloxacin. The complexation of the antibiotic with SBE-*β*-CD increased the encapsulation efficacy; the synthesized NPs were ensured to interact with the eye surface thanks to their high positive zeta potential. The researchers also reported that the free antibiotic was two times weaker that the NPs against the tested Gram-negative pathogens: *E. coli*, *P. aeruginosa*, and *S. aureus* [84].

Levofloxacin benefits from its chemical and physical interactions with natural macromolecules, each one offering a different benefit such as increased encapsulation efficiency, drug loading, surface interactions, and antimicrobial properties, just to name a few. Other than chitosan NPs, Table 4. presents a few of the macromolecules used for the synthesis and functionalization of levofloxacin NPs. Each polymer comes with its own advantages, each one being effective in certain presented scenarios.

As greener alternatives are becoming much more popular, biomolecules such as alginate, gelatin, agar, and starch are employed for the synthesis of biodegradable NPs. The advantage of using naturally occurring macromolecules for the synthesis of functional antibiotic alternatives is their increased biocompatibility compared to synthetic hydrocarbon polymers as well as their lower toxicity, being easily metabolized and eliminated [85,86,87].

Other advantages to using biopolymers compared to synthetic ones are their intrinsic antioxidant and antibacterial properties, being ideal against resistant pathogens and ROS [88].

**Table 4:** Commonly used polymers for the synthesis of Levofloxacin NPs and the benefits of using them.

Polymer	Key Properties	References
Chitosan	- Biocompatible, biodegradable - Enhances mucoadhesion and prolonged drug release - Improves antimicrobial activity against biofilms and MDR bacteria	[89,90]
SBE-β-CD	- Increases drug solubility - Improves stability - Enhances bioavailability and sustained release	[84,91,92,93]
Alginate	- Natural polymer with biocompatibility - Provides controlled drug release - Gel-forming properties aid in wound healing	[94,95]
PLGA (Poly(lactic-co-glycolic acid))	- FDA-approved, biodegradable - Long circulation time - Controlled and sustained release	[96]
Gelatin	- Biodegradable and biocompatible - Non-toxic with good bioavailability - Improves drug stability and targeting	[97,98,99]
PCL (Polycaprolactone)	- Biodegradable synthetic polymer - Provides long-term sustained release - Low toxicity, improves drug loading efficiency	[100,101,102]

#### 3.2.2. Phosphorus-Containing Antibiotic/Polymer Composite

Phosphorus atoms have an increased biological activity and are a crucial part of physiological processes.

Phosphorus antibiotics are substances derived from the following:

- Other antibiotic classes such as β-lactamases (Tazobactam phosphate);
- Cyclical polypeptides (Bacitracin, Daptomycin, and Polymyxin B);
- Amino acids (Aminomethylphosphonic acid and N,N-Bis(2-Aminoethyl)phosphonic acid).

In general, these antibacterial substances contain one or more phosphorous atoms in different forms. Polypeptides like the ones mentioned above have, in most cases, the hydroxyl groups of serine or threonine residues phosphorylated to maximize their antimicrobial and biological potential [103]. Table 5 presents a review of the phosphorous-containing antimicrobial substances reported in the recent literature, as well as their main mechanism of action and their chemical structure.

**Table 5:** Phosphorous-containing antimicrobial substances, their structure, and mechanism of action.

Name	Structure Description	Mechanism of Action	References
Fosfomycin	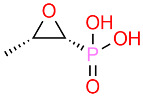	Inhibits bacterial cell wall synthesis by targeting murA.	[104,105,106]
Bacitracin	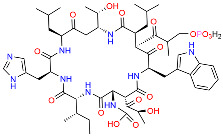	Interferes with cell wall synthesis in Gram-positive bacteria.	[107,108]
Tazobactam	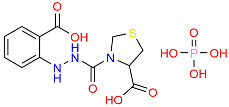	Inhibits β-lactamase enzymes, enhancing the effectiveness of β-lactam antibiotics.	[109,110,111]
Cyclic phosphonates	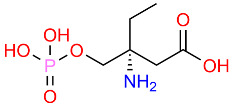	Potential mechanisms like fosfomycin, targeting cell wall synthesis.	[112,113,114]
Amino phosphonates	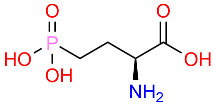	The specific antimicrobial mechanisms can vary.	[115]

Zhu et al., 2023 worked on the synthesis of synergistic fosfomycin liposomes absorbed by polymyxin B, after which the researchers tested the product both in vitro and in vivo on *Acinetobacter baumann*. It was found that the modified liposomes showed and increased antibacterial effect compared to the control and the liposomes were not cytotoxic [116].

It is known that polymyxin B is nephrotoxic, but by the functionalization with liposomes, this disadvantage was lower compared to the polymyxin B control, indicating the detoxifying properties of liposomes [117,118].

Daptomycin has also been used for the synthesis of mucoadhesive NPs for treating endophthalmitis caused by Gram-positive bacteria (MRSA, *S. epidermidis*, *Staphylococcus lugdunensis*, *haemolyticus*, *hominis*, and *faecalis*). The NPs were made using chitosan and sodium tripolyphosphate by ionotropic gelation. The final NPs measured about 200 nm and were very efficient at encapsulating daptomycin (maximum 97%); in vitro tests showed that all the antibiotic was released over a period of 4 h. Stability tests revealed that the NPs were not degraded by lysozyme and that mucin had a positive impact on mucoadhesiveness, making them ideal for the treatment of ocular bacterial infections [119].

Costa et al., 2015 synthesized chitosan and sodium alginate NPs loaded with daptomycin for the treatment of ocular infections, testing the NPs on methicillin-susceptible *Staphylococcus aureus* (MSSA), MRSA, *S. epidermidis*, *Staphylococcus capitis*, *Staphylococcus hominis*, *Staphylococcus lugdunensis*, *Staphylococcus haemolyticus*, and *Staphylococcus warneri*. The final NPs measured about 380–420 nm and were very efficient at loading the antibiotic (maximum 92%); however, after a period of 4 h in vitro, only a maximum of 12% of the encapsulated daptomycin was released. The researchers found this peculiar and suggested that the NPs could be used for carrier systems over prolonged periods of time, making the administration of the antibiotic more efficient [120].

In another study, researchers worked to synthesize PCL NPs encapsulating daptomycin and tested them on Gram-positive bacteria such as MRSA and PIA-positive *S. epidermidis*. The encapsulation efficiency of these NPs was around 83% with slight variations, a similarity with previous tests is seen here where the daptomycin release took place over the span of 72 h, with a percentage of around 10.4%. Daptomycin NPs were superior in antibacterial effect compared to their vancomycin counter parts, being able to impede biofilm development and decrease its mass. The daptomycin NPs also proved compatible with cell lines such as L929 (mouse fibroblasts) and MG63 (human osteoblast-like cells) [121].

## 4. Preparation Process—Literature Review

A thorough literature search was conducted for the writing of this paper; international public databases such as Google Scholar, Web of Science, PubMed, and Scopus were used to acquire the necessary data. During the search, keywords such as “antibiotic-resistance”, “superbugs”, “antibiotics”, “biosynthesis”, “polymers”, and “antibiotic alternatives” were used to ensure the search of relevant publications to our study.

**Figure 6:**
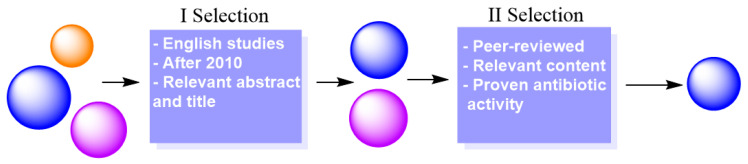
The two-step selection process that was made particularly for the theme of this review to extract only relevant information.

After this search, the publications were subjected to a two-step selection process, presented in Figure 6. Firstly, the articles were chosen based on the information presented in the title and abstract. At this point, the year of publication was important, selecting only articles published after 2010, with exceptions for representative works. The year of publication is a crucial parameter as it ensures that the information is new and updated as technologies progressed.

Secondly, the articles were subjected to another more rigorous separation where works that were not peer-reviewed or did not present any concise information on the antibacterial action of polymers were eliminated. In the end, all the articles that were left after this process were added to a citation database.

Data extraction focused on key details such as the characteristics of several types and synthesized polymers and their use as is or in NP encapsulation for delivering antibiotics. Data regarding the antibacterial activity of the synthesized polymers was gathered from both in vitro and in vivo studies presenting relevant testing methods and techniques, making sure they complied with the ethical norms. The mechanism of action of these polymers was also important to the study as different synthesis products can cause the disruption of the cell wall, inhibiting the formation of biofilm, or they can interfere with different enzymes involved in antibiotic resistance.

The present manuscript was structured to highlight the distinctive characteristics of alternative polymer-based antibiotics against resistant pathogens. The literature review opens with a general synthesis of antibacterial polymers, which is followed by other important aspects regarding their antimicrobial profile and pharmacodynamical properties.

## 5. Future Directions

The persistent epidemic of antibiotic resistance constitutes a significant concern to public health, since drug-resistant illnesses result in prolonged hospitalizations, heightened death rates, and rising healthcare expenditures worldwide. Traditional antibiotics are becoming less effective due to extensive abuse and overuse, enabling bacteria to adapt and establish formidable defensive mechanisms, including biofilms that protect bacterial colonies from treatment and efflux pumps that actively remove drugs before they can exert their effects. The dilemma is exacerbated by a declining pipeline of new antibiotics, as several pharmaceutical firms are diverting their focus from antibiotic research owing to elevated development costs and minimal financial returns, hence further constraining our alternatives against these “superbugs”.

Polymer-based alternatives provide a potential option; yet, they encounter existing obstacles that restrict their practical use. Challenges encompass the want for accuracy in targeting particular germs without adversely affecting human cells and reducing toxicity, alongside the scalability and economic viability of polymeric materials for extensive medicinal use. The inhibition of biofilm necessitates polymers capable of both obstructing bacterial adhesion and infiltrating the pre-existing biofilms on medical devices, a challenging task that demands extensive testing and modification. Moreover, whereas nanoparticle–polymer systems exhibit promise for the efficient delivery of antibiotics, preventing these particles from eliciting immune responses or accumulating in organs is a significant problem. Resolving these challenges through multidisciplinary research and regulatory assistance is crucial for rendering polymeric antibiotic alternatives a feasible option in the near future. For example, the blood–brain barrier is a common obstacle in the administration of antibiotics in nervous system infections and targeting is crucial so as to avoid possible neurological and psychological side-effects [122].

The integration of green synthesis and sustainable practices in polymer fabrication also holds significant promise. Biodegradable polymers such as chitosan and starch exhibit inherent antimicrobial properties and offer an eco-friendly alternative to traditional treatments. Future research should focus on refining the use of these natural polymers, possibly by integrating halogen-containing compounds for enhanced antimicrobial action. Chitosan–iodine complexes, for instance, have shown great potential, but the field remains relatively unexplored. Further studies on their mechanism of action and long-term stability will pave the way for their use in clinical settings.

Future research is needed for the in vivo study of these antibiotic polymers and NPs, seeing how nervous system infections often have long-term psychological and neurological effects on patients. In afflictions like encephalitis and meningitis, fluid buildup can lead to the increase in intercranial pressure and even possible changes in the behavior or personality of individuals. This is why psychiatric evaluation after nervous system infections is so important, and future studies can give us indications on more effective drugs that can cross the blood–brain barrier and have fewer side-effects.

Another promising direction lies in the field of nanotechnology, where NPs can be utilized for more efficient drug delivery. Polymers could serve as carriers for these NPs, enhancing both the specificity and effectiveness of antibiotic treatment. Chitosan-based NPs, for example, could encapsulate antibiotics, protecting them from degradation while delivering them directly to the bacterial cells. NPs functionalized with antimicrobial peptides or other natural compounds could be developed to create more potent antimicrobial formulations that target resistant pathogens without promoting further resistance.

It is also crucial to investigate novel classes of antibiotics that use unconventional mechanisms, sidestepping traditional bacterial resistance pathways. One option is the continued exploration of antimicrobial peptides that can directly disrupt bacterial cell membranes. Understanding how to enhance their stability and integration into synthetic polymers is vital for developing next-generation antimicrobials. Enhancing the bioavailability and safety of these compounds in vivo should be a key focus, alongside studies into their pharmacodynamics and pharmacokinetics.

Interdisciplinary collaboration between microbiologists, pharmacists, material scientists, and clinicians will be essential in moving these innovations forward. To optimize the efficacy of antimicrobial polymers, researchers must further investigate their interaction with different bacterial species, including their ability to overcome the adaptive resistance mechanisms that bacteria develop.

Overall, these future research directions offer a comprehensive strategy to combat antibiotic resistance. By combining the power of polymers, NPs, and innovative synthesis methods, it is possible to create therapies that ignore bacterial resistance mechanisms, offering hope for a future without superbugs.

## 6. Conclusions

In their pursuance of inventive antimicrobial interventions, polymers, particularly biopolymers such as chitosan and PCL, have established themselves as transformative materials. Chitosan, a chitin derivative, demonstrates a unique combination of features that augment its antimicrobial efficacy, chiefly through its distinctive interaction with microbial cell membranes, allowing it to enter and destroy them. This mechanism enables chitosan to effectively combat a diverse range of microorganisms, including both Gram-positive and Gram-negative bacteria, positioning it as a highly adaptable candidate for advanced drug delivery systems. These systems not only improve therapeutic results but also reduce the negative effects associated with traditional antibiotic doses, establishing chitosan as an essential asset in contemporary medicinal chemistry.

The current investigation of polymers including phosphonium, ammonium, and halogens illustrates the versatility and significant antibacterial capabilities inherent in polymeric materials. Particularly as a result of their capacity to penetrate membranes and their capacity to deconstruct biofilms, these polymers demonstrate exceptional antibacterial efficacy. Biofilms, which provide robust protection for bacterial colonies in clinical settings, frequently impede conventional therapies. By targeting and dissolving these protective biofilms, these polymers provide an innovative strategy to overcome the antibiotic resistance that persistently challenges global healthcare systems.

Ultimately, the critical role that these materials play in contemporary infection control strategies is underscored by the development of versatile biopolymers, such as chitosan and halogen- or quaternary salt-containing polymers, which have been made possible by advancements in polymer science. Their intrinsic antibacterial characteristics and improvements in medication delivery are transforming therapeutic and preventative strategies. With advancements in research, polymers are set to play a pivotal role in combating infections, particularly those caused by resistant bacterial strains, signaling a significant transformation in antimicrobial innovation.

## Figures and Tables

**Table 1:** Chitosan antibacterial properties and their mechanism of action.

Mechanism of Action	Description	References
Membrane disruption	- Interacts with the negatively charged bacterial membrane, increasing its permeability, and leading to the leakage of intracellular contents and cell death.	[40]
Inhibition of DNA transcription and replication	- Penetrates bacterial cells and binds to DNA, interfering with transcription and replication, and inhibiting growth.	[41]
Chelation of metallic ions	- Binds to divalent metal ions like Ca^2+^ and Mg^2+^, which are critical for bacterial enzyme function, leading to metabolic disruption.	[42]
Biofilm disruption and inhibition	- Disrupts mature biofilms and prevents bacterial adhesion, thus inhibiting biofilm formation and growth.	[43]
Osmotic imbalance	- Interaction of chitosan with bacterial cell walls causes structural damage, leading to osmotic imbalance and eventual cell lysis.	[44]
Interference with protein synthesis	- Interferes with ribosomal function, impairing protein synthesis, which leads to inhibition of bacterial growth and survival.	[45]
Increased reactive oxygen species (ROS) production	- Induces the production of ROS in bacterial cells, leading to oxidative stress, lipid peroxidation, and cell death.	[46]
Interaction with bacterial surface proteins	- Binds to surface proteins on bacteria, altering cellular processes such as nutrient uptake and membrane stability.	[47]
Electrostatic interaction with the outer membrane	- Chitosan’s positive charge attracts the negatively charged components of bacterial outer membranes, disrupting the membrane integrity.	[48]

**Table 2:** The percentage of released iodine from the HPCGH-PVP-I_2_-M membrane over a period of 200 min.

Temperature (°C)	Released Iodine (%)
20	3.8
30	5.8
37	6.7
45	9.8
50	12.5

**Table 3:** Thermal stability of phosphonium polymers depending on the length of the alkyl chain.

Polymer	T_g_ (°C)
Poly[trimethyl-(4-vinylbenzyl)phosphonium chloride]	284
Poly[triethyl-(4-vinylbenzyl)phosphonium chloride]	240
Poly[tripropyl-(4-vinylbenzyl)phosphonium chloride]	195
Poly[tributyl-(4-vinylbenzyl)phosphonium chloride]	177
